# Effective Detection of Cloud Masks in Remote Sensing Images

**DOI:** 10.3390/s24237730

**Published:** 2024-12-03

**Authors:** Yichen Cui, Hong Shen, Chan-Tong Lam

**Affiliations:** 1 Faculty of Applied Sciences, Macao Polytechnic University, Macao SAR, China; p2316993@mpu.edu.mo (Y.C.); ctlam@mpu.edu.mo (C.-T.L.); 2 School of Engineering and Technology, Central Queensland University, Rockhampton 4701, Australia

**Keywords:** cloud mask detection, U-shaped structure, dual up-sampling module, context information full flow

## Abstract

Effective detection of the contours of cloud masks and estimation of their distribution can be of practical help in studying weather changes and natural disasters. Existing deep learning methods are unable to extract the edges of clouds and backgrounds in a refined manner when detecting cloud masks (shadows) due to their unpredictable patterns, and they are also unable to accurately identify small targets such as thin and broken clouds. For these problems, we propose MDU-Net, a multiscale dual up-sampling segmentation network based on an encoder–decoder–decoder. The model uses an improved residual module to capture the multi-scale features of clouds more effectively. MDU-Net first extracts the feature maps using four residual modules at different scales, and then sends them to the context information full flow module for the first up-sampling. This operation refines the edges of clouds and shadows, enhancing the detection performance. Subsequently, the second up-sampling module concatenates feature map channels to fuse contextual spatial information, which effectively reduces the false detection rate of unpredictable targets hidden in cloud shadows. On a self-made cloud and cloud shadow dataset based on the Landsat8 satellite, MDU-Net achieves scores of 95.61% in PA and 84.97% in MIOU, outperforming other models in both metrics and result images. Additionally, we conduct experiments to test the model’s generalization capability on the landcover.ai dataset to show that it also achieves excellent performance in the visualization results.

## 1. Introduction

From an area perspective, clouds and cloud shadows appear in 67% of the remote sensing images. Cloud shadow coverage can negatively affect the in-depth study of remote sensing images. Cloud masks severely constrain the ability to extract key feature information including agriculture, forests, cities, etc., from remote sensing images. Identifying cloud shadows and locating them accurately are helpful for weather forecast tracking and obtaining meteorological information, which provides an indelible contribution to disaster prevention and reduction.

With the development of deep learning, many semantic segmentation models have widely been used in image processing. FCN [1] introduced the basic encoder–decoder architecture in semantic segmentation for the first time, which was widely adopted by many subsequent methods. U-Net [2] implemented channel concatenation of feature maps from neighboring layers, which nicely complemented the contextual information and thus solved the segmentation task at the cellular level. To achieve higher accuracy, PSPNet [3] used a pyramid pooling module to aggregate global context information.

In recent years, many new learning paradigms have emerged in the field of semantic segmentation [4,5,6] and used the common CNN framework to address segmentation problems from various angles. Liu et al. [7] improved Deeplabv3 in a lightweight manner, thereby integrating the low-level features, reducing the computational complexity, and refining the object edges. Sung et al. [8] proposed a contrastive learning-based semantic segmentation method, allowing for context to acquire multi-scale feature fusion through contrastive learning, thus achieving better discriminative capabilities. With the emergence of large models, Transformers have also been widely applied in semantic segmentation [9].

However, numerous challenges emerged when these models were applied to cloud-shadow detection tasks. The FCN model, being unidirectional, did not account for the integration of contextual information, which made it challenging to address issues related to cloud shadow occlusion and edge segmentation. U-Net and SegNet [10], relying solely on standard 3 × 3 convolution operations, resulted in a limited receptive field within the extracted feature maps. This led to an overemphasis on local information and a neglect of global information, which is crucial for accurately pinpointing small targets such as thin and fragmented clouds. Additionally, snow, which shares similar color and spectral characteristics with clouds, was prone to being misidentified as continuous cloud cover. Transformers heavily utilize the multi-head attention mechanism, which results in a large number of model parameters and a high computational complexity, affecting the practical deployment of the model.

To solve the above problems, we propose a Multiscale Dual Upsampling Network called MDU-Net for cloud-shadow detection that can refine the edges of a cloud shadow, accurately locate thin and broken cloud masks, and also deal with special scenes such as saline fields in shadows. The main contents of this article are as follows:

- We employ ResNet34 [11] as the backbone network, utilizing residual connections to fuse the original features with the high-level features. This integration ensures that the feature maps encompass not only global information but also a wealth of detailed information. The model pays more attention to the spatial and classification features of cloud shadows, achieving refined edge segmentation.
- In the decoder, we design a dual up-sampling module. The first up-sampling module achieves full flow of contextual information between each neighboring layer, which ensures that the feature maps do not focus too much on the local regions of the same layer, and replenish the global information lost by multiple convolutions. This not only strengthens the model’s advantage of accurate spatial localization of small targets such as thin and broken clouds, but also reduces the detection interference of cloud layers caused by lighting conditions. The second up-sampling module is used to directly up-sample the deep feature maps before channel concatenation to retain the original semantic information. This information helps the surface salinity, snow, and clouds to be differentiated by categories in terms of color texture and spectral features.

## 2. Related Work

There have been two main types of mainstream directions in cloud masking detection methods in the last two decades.

### 2.1. Machine Learning Models Based on Classification

As the resolution and clarity of remotely sensed imagery increased dramatically, traditional threshold segmentation could no longer meet the accuracy requirements of scientific research [12]. Thus, machine learning algorithms enabled the detection of clouds and cloud shadows by extracting reflective, textural, and local statistical features.

Fu et al. [13] introduced random forests to the cloud detection task, which better handled complex nonlinear dependencies between multiple feature types. Liu et al. [14] introduced daytime and nighttime machine learning algorithms, adding surface variables in a binary manner to distinguish cases including solar spectral band observations, thus enhancing the accuracy of cloud pixel detection. Ibrahim et al. [15] employed a supervised SVM to distinguish clear pixels such as clouds and cloud shadows. Due to the variation in the angle of direct solar illumination, there are significant errors in the cloud shadow images captured by the sensor; the authors improved the identification results from a geometric perspective. Miroszewski et al. [16] designed support vector machines (SVMs) with quantum kernels, where pixels were mapped to the Hilbert space using a set of parameterized quantum features. On multispectral satellite images, the hybrid SVM effectively addressed the problem of cloud detection. Gao et al. [17] analyzed common machine learning classification methods and, by combining S2cloudless with random forest, successfully resolved the issue of cloud masking in Sentinel-2 imagery, thereby ensuring the accurate mapping of paddy rice in southern China.

Although the machine learning model exceeded the traditional thresholding method in classification accuracy, its pre-data pre-processing required a large number of hand-designed image features, and was unable to deal with more complex scenes with more advanced semantic information [18].

### 2.2. Deep Learning Algorithms Based on Semantic Segmentation

Since 2012, classic backbone networks such as AlexNet [19], VGG [20], and ResNet have been applied to semantic segmentation tasks. Cloud and shadow detection essentially involves separating clouds and shadows from the background, which aligns with the definition of pixel-level classification in semantic segmentation [21].

Li et al. [22] employed a hierarchical Transformer to extract features of clouds and shadows (CS). In the decoder, several simple multi-layer perceptrons (MLPs) were used to fuse multi-scale feature maps to achieve pixel classification. Dong et al. [23] introduced a multi-level cloud detection network that effectively identifed the edges of thin clouds and broken clouds. Zhang et al. [24] proposed a reconfigurable network driven by multi-task learning (MTDR-Net), which facilitated global information interaction and simplified the model without losing its structural integrity.

Zhang et al. [25] proposed a refined segmentation network for clouds and cloud shadows, utilizing a multi-scale global attention module to enhance channel and spatial information, thereby reducing the impact of ground objects and other noises and improving detection accuracy. Chen et al. [26] introduced a multi-scale strip feature attention network (MSFANet), which incorporated a deep-layer multi-scale pooling attention module to extract multi-scale contextual semantic and spatial information. The boundary detail feature perception module leveraged skip connections to perform feature exploration in both the height and width dimensions of the feature map, restoring the boundary detail information of the detection targets. Gu et al. [27] proposed a multi-path multi-scale attention (MMA) network, which introduced multi-scale overlapping patches and a multi-path pyramid vision transformer to achieve effective aggregation of fine and coarse features at the same feature level. These modules focused on important information in the image, removed network noise, and improved the detection accuracy of small targets.

The above cloud detection models previously discussed seldom considered the interference from clear backgrounds formed under varying lighting conditions on the detection of clouds [28]. The shadow pixels of clouds, exhibiting darker tones, were particularly susceptible to misidentification with backgrounds such as black soil [29].

## 3. Methods

**Figure 1:**
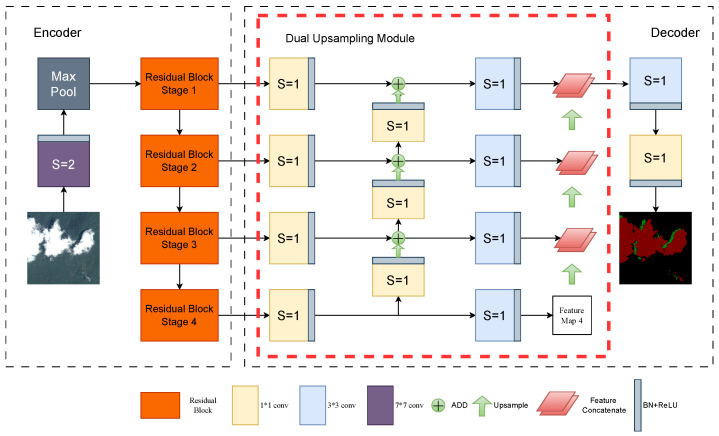
The structure of MDU-Net.

Earlier U-shaped structures introduced for cloud detection tasks were prone to lose spatial information when extracting cloud shadows, resulting in blurred edges. For these problems, we propose a multiscale dual up-sampling segmentation network called MDU-Net that adopts an encoder–decoder–decoder framework and has the overall architecture shown in Figure 1.

### 3.1. Overview

The overall structure of MDU-Net is encoding–decoding–decoding. The input image with a resolution of 224×224×3 is first down-sampled to 1/2 by a large 7×7 convolutional kernel with a step size of 2 and a maximum pooling layer to reduce the amount of parameter computation.

Since cloud detection is a multi-class segmentation task, we use a residual structure in the down-sampling stage of the encoder. The ordinary residual module with a step size of 1 extracts the features of clouds and shadows, while the down-sampling residual module with a step size of 2 acquires the deeper information to obtain the rich categories information of clouds and shadows. The U-shaped structure of the network is set to be four down-samplings as well as retaining the output of the feature maps in the four phases, which ensures that the resolution of the feature maps is neither too large nor too small. The residual connection of the four stages effectively extracts the multi-scale information of clouds and shadows, ensures the network depth, and refines the edges of clouds and shadows.

Since thin clouds can be cluttered with objects with high surface reflectivity, and broken clouds are often scattered throughout the image as small targets, the up-sampling method of the ordinary U-Net in the decoder part is to directly splice the channels of the feature maps of the same layer without any processing of the feature maps output from the encoder. Different from the design idea of traditional U-Net, before the fusion of the same-layer feature maps in the first up-sampling, we add 1×1 convolution to enhance the extraction of the category information of the thin clouds. The first up-sampling not only achieves the fusion of contextual information for spatial localization of the broken clouds, but also provides richer multi-scale information for the second up-sampling.

After the feature extraction in the first round of up-sampling, we add a 3×3 convolution module in the second round of up-sampling, whose output feature maps are continuously spliced and fused with the feature maps of the neighboring layers that have been up-sampled, and finally fed into the redesigned segmentation module to achieve the cloud detection targets. Compared to the increasingly complex segmentation models, the overall structure of MDU-Net is relatively simple and straightforward, and achieves better detection accuracy on synthetical datasets. The modules and design principles of our proposed network are demonstrated in detail in the following subsections.

### 3.2. Residual Module

The feature information extracted by the backbone network greatly affects the final accuracy of the segmentation task. However, the ordinary convolution used by U-Net causes the network to encounter a gradient bottleneck during backpropagation through the loss function. The reason for this is that ordinary convolution continuously captures high-level feature information such as cloud contours, but ignores low-level features such as the color and texture of the ground surface. Therefore, in this paper, we consider the mainstream method of preserving features on backbone networks: residual structure.

**Figure 2:**
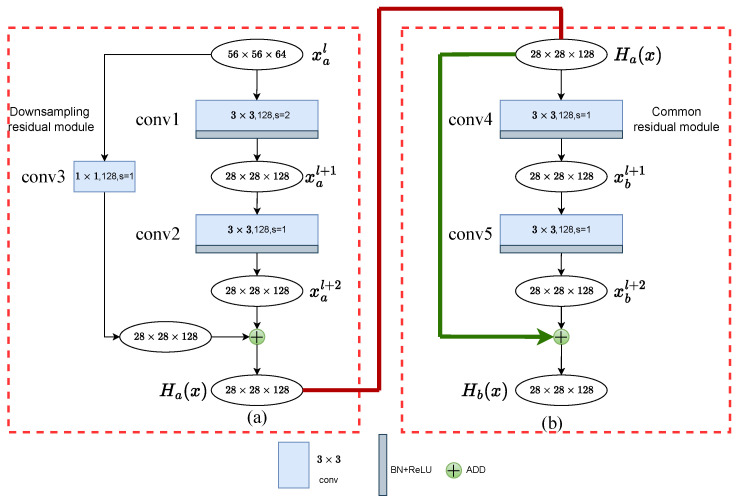
The structure of the residual module. (**a**) Downsampling Residual Module. (**b**) Standard Residual Module.

As the depth of neural network layers increases, the accuracy of the model tends to degrade. He et al. [11] designed the residual block and introduced shortcut connections between layers to balance linear and nonlinear transformations. As shown in Figure 2, a residual block consists of two modes: the left side represents the down-sampling mode, and the right side represents the standard mode. In the down-sampling residual block (Figure 2a), the feature map $x_{a}^{l}$ is first fed into a convolutional layer with a stride of 2 to reduce the resolution, as given by the formula:

(1) $$x_{a}^{l+1}=RELU_{BN}\left(W_{a}^{l+1}\times \left(x_{a}^{l}\right)+b_{a}^{l+1}\right)$$

Here, elements with subscript *a* indicate data processed within the down-sampling residual block. $x_{a}^{l}$ represents the original input feature map, assumed to have dimensions 56×56×64. $W_{a}^{l+1}$ represents the weight parameters of the first convolutional layer (conv1) in the down-sampling block, and $b_{a}^{l+1}$ represents the bias layer of this convolutional block. The output of this convolutional layer is $x_{a}^{l+1}$, with dimensions reduced to 28×28×128.

Following the down-sampling, the block further employs a convolutional layer (conv2) to extract abstract information about cloud and shadow features without altering the resolution, thus setting the stride to 1. The formula is as follows:

(2) $$x_{a}^{l+2}=RELU_{BN}\left(W_{a}^{l+2}\times \left(x_{a}^{l+1}\right)+b_{a}^{l+2}\right)$$

Here, $x_{a}^{l+2}$ denotes the output feature map of conv2, maintaining the dimensions 28×28×128. $W_{a}^{l+2}$ and $b_{a}^{l+2}$ represent the weight parameters and bias layer of conv2, respectively. Thus, the formula for the downsampling residual block is

(3) $$H_{a}\left(x\right)=f_{s=1}^{3\times 3}\left(f_{s=2}^{3\times 3}\left(x_{a}^{l}\right)\right)+f^{1\times 1}\left(x_{a}^{l}\right),$$

where the residual refers to the difference between the observed value and the estimated value. $H_{a}\left(x\right)$ is the observed value with dimensions 28×28×128, and $x_{a}^{l}$ is the estimated value. Since conv1 down-samples $x_{a}^{l}$, the identity mapping must also down-sample the input feature $x_{a}^{l}$ to maintain consistency, which is achieved through a 1×1 convolution, denoted by $f^{1\times 1}\left(\right)$, forming the shortcut connection. $f_{s=2}^{3\times 3}\left(\right)$ and $f_{s=1}^{3\times 3}\left(\right)$ represent the convolution operations with strides of 2 and 1, respectively, each composed of a 3×3 convolution, a BN layer, and a ReLU activation function.

As illustrated in Figure 2b, the observed value output $H_{a}\left(x\right)$ from the down-sampling residual block serves as the input to the standard residual block. The specific formulas are as follows:

(4) $$x_{b}^{l+1}=RELU_{BN}\left(W_{b}^{l+1}\times \left(H_{a}\left(x\right)\right)+b_{b}^{l+1}\right)$$

(5) $$x_{b}^{l+2}=RELU_{BN}\left(W_{b}^{l+2}\times \left(x_{b}^{l+1}\right)+b_{b}^{l+2}\right)$$

(6) $$H_{b}\left(x\right)=f_{s=1}^{3\times 3}\left(f_{s=1}^{3\times 3}\left(H_{a}\left(x\right)\right)\right)+H_{a}\left(x\right)$$

In these equations, elements with subscript *b* indicate data processed within the standard residual block. Unlike the down-sampling residual block, the standard residual block only needs to extract fine-grained features of clouds and shadows while maintaining the resolution; hence, the stride in conv4 is set to 1, denoted by $f_{s=1}^{3\times 3}\left(\right)$. To maintain the resolution, the shortcut connection directly adds the original input $H_{a}\left(x\right)$ to the output of conv5 to produce the final output of the residual block $H_{b}\left(x\right)$. Additionally, the remaining parts of the standard residual block follow the same operations as those in the down-sampling residual block, with all convolutional layers producing feature maps of dimensions 28×28×128.

The skip connections in residual blocks excel at integrating high-level spatial features with low-level information such as color and texture, thereby enhancing the model’s focus on both spatial and categorical information of clouds and shadows, and enabling precise detection at the interface between cloud shadows and the background.

### 3.3. Dual Up-Sampling Module

The classical U-shaped model will directly splice the feature map of the *l* layer encoder with the output structure of the l+1 layer decoder after up-sampling, and then feed it into the convolution module and repeat the above operation until it is fed into the segmentation task header to obtain the final result. The above process is the contextual information fusion, where contextual information refers to the correlation between different pixels. In a rich model, contextual information can be achieved not only by widening the network sensory field method, but also by the multi-scale fusion of feature maps at different levels. For semantic segmentation tasks, the extraction of contextual information greatly affects the recognition of small targets.

Therefore, after UNet was proposed, many medical image networks set up various new modules between the encoder and the decoder to achieve the extraction of contextual information. Before the feature maps concatenation, MultiResUNet [30] proposed a residual path module, which performed 3×3 convolution and residual operations on features for many times to reduce the semantic gap between the low-level features of the encoder and the high-level features of the decoder. However, the module still only extracted the context relationship between the feature maps of adjacent layers, and did not realize the real context information circulation.

**Figure 3:**
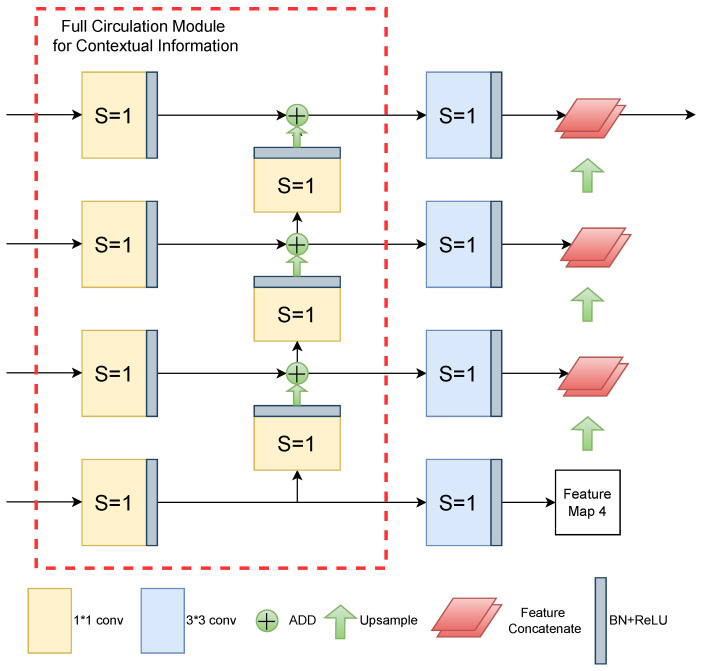
The structure of dual up-sampling module.

The common U-shaped structure of the decoder is just a single layer structure in the up-sampling stage, and in order to ensure that the feature map achieves the full flow of contextual information in the decoder section, we design the decoder as a dual up-sampling module. As shown in Figure 3, in the left column of the decoder part, we design the context information full circulation module. Since the backbone network of the encoder is divided into four stages, we multiply the number of convolution kernels of each neighboring stage to output the feature map according to the criterion of the residual module. Let us take the feature map $X_{2}$ output from stage 2 as an example whose resolution is 28×28×128. Firstly, we use 128 1×1 convolutional kernels with BN and ReLU layers to add nonlinear excitation to the feature map to improve the network’s expressive ability, and the output feature map $X_{21}$ is still 28×28×128. Then, we expand the resolution of the feature map $X_{21}$ with $X_{21}$ from stage 3 after up-sampling and bilinear interpolation, and the resolution of the feature map $X_{21}$ is still 28×28×128. The feature map from stage 3 after up-sampling bilinear interpolation to expand the resolution is then summed with $X_{21}$ to get $X_{22}$.

The full flow of contextual information between each neighboring layer ensures that the feature maps extracted by the model will not be too focused on the local region of the same layer, which supplements the global information lost in the previous problem. This not only strengthens the model’s advantage of accurate spatial localization of small targets such as thin and broken clouds, but also reduces the detection interference of cloud layers due to lighting conditions, making it possible to distinguish cloud shadows with pixel values close to (0, 0, 0) from backgrounds such as black land. Therefore, $X_{22}$ will not only be output to the decoder in the same layer for final up-sampling to achieve feature map reduction, but also accept the feature map fusion from stage 3, while the model fuses $X_{22}$ with the output feature map of stage 1 again, so that the contextual information full flow module is completed.

The feature results extracted from stage 4 are ultimately retained until they are fused with the stage 1 feature map. The number of feature map channels will be doubled after channel concatenating, which will generate a large number of parameters and consume memory during model training. Therefore, for the first up-sampling feature fusion, we adopt the method of addition to strengthen the weight parameters for effectively identifying small targets such as thin clouds and broken clouds.

During the up-sampling stage, the common U-shaped structure concatenates adjacent feature maps. This cycle of up-sampling and convolution operations increases the model’s parameters and computational load, and can lead to model degradation. It does not effectively preserve semantic information such as saline–alkali land and ground snow, nor can it distinguish between the color and spectral characteristics of clouds.

MDU-Net uses only one 3×3 convolution operation for each layer for the second up-sampling feature fusion. Since the number of feature map channels output from stage1–4 is [64, 128, 256, 512], if the number of feature map channels is kept unchanged, this will lead to redundancy of information, and will not be conducive to the enhancement of the recognition ability of similar background features. Therefore, the number of 3×3 convolution kernels of MDU-Net in this part are all set to 128, and then the output feature maps are not subjected to other convolution or BN and RELU operations. This kind of feature maps not only retain the original color texture information of clouds and shadows, but also carry similar semantic information of spectral features such as saline ground, which has a considerable improvement in the accuracy of the cloud detection task in some special scenarios, which is detailed in the experimental part of this paper.

Finally, due to four channels concatenating, the number of feature map channels reaches 512. In order not to let the information of the spliced feature map be lost at the end, we still set 512 3×3 convolution kernels to keep the fused features as much as possible, while this paper is a three-class segmentation task, so we finally add three 1×1 convolution kernels to output the segmentation map. Next, the effectiveness of MDU-Net is verified by experimental results.

## 4. Experimental Analysis and Results

### 4.1. Dataset

**Figure 4:**
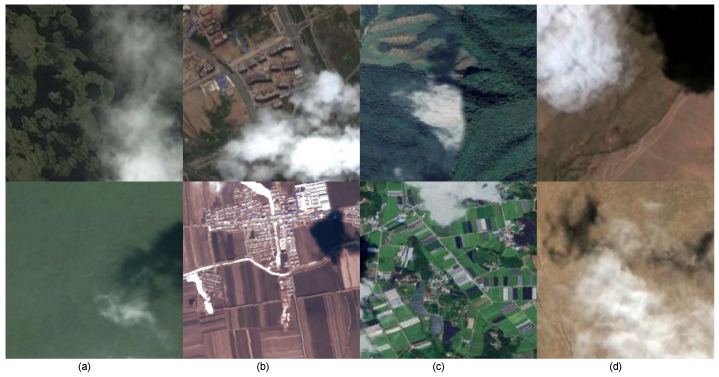
The backgrounds of self-made dataset. (**a**) Water areas. (**b**) Cities. (**c**) Vegetation. (**d**) Deserts.

The cloud and cloud shadow datasets used in this paper are mainly collected from Google Earth and the U.S. LandSat8 [31,32]. This dataset is mainly from Chinese mainland, the minimum ground distance of remote sensing image is 30 m, and the view height is 15 km. The imager of LandSat8 has 9 bands. In this paper, spectral bands 2, 3, and 4 are combined into natural true color satellite images. Finally, we cut its resolution to 224×224. To ensure the diversity of background samples, this dataset collected scenes such as water, forests, farmland, cities, deserts, plains, and hills. There are 2557 224×224 RGB images in the dataset, of which 2045 are used as training sets and 512 are used as verification sets. As shown in Figure 4, we label the dataset and divide it into three categories: cloud, cloud shadow, and background.

### 4.2. Training Details

MDU-Net is implemented based on PyTorch (2.0.1), cuda (11.8.0), and cudnn (8.9). We set the initial learning rate to 0.001 and the learning rate adjustment multiplier is 0.95. We set the step size to 3, and the Adam optimizer is used. Due to the limited experimental conditions, the hardware device used in this task is NVIDIA GeForce RTX 2080TI 11G (Santa Clara, CA, USA). The batch size is set to 8 only, and the epoch is set to 100. We use the StepLR mechanism to adjust the learning rate, with the specific formula as follows:

(7) $$lr=lr\times \gamma^{\left\lfloor \frac{epoch}{step_{size}}\right\rfloor }$$

Here, lr represents the current learning rate. γ is the decay factor of the learning rate, typically a positive number less than 1. step_size is the period for adjusting the learning rate, i.e., the number of epochs after which the learning rate is multiplied by γ. epoch is the current training epoch. ⌊x⌋ denotes the floor function, which rounds down to the nearest integer.

We choose the cross-entropy loss function to calculate the sample error of the model. In practical applications, we usually handle batch samples. Assuming the batch size is *N*, the total cross-entropy loss *L* is given by

(8) $$L=-\frac{1}{N}\sum_{n=1}^{N}\sum_{i=1}^{C}y_{ni}\log\left({\hat{y}}_{ni}\right)$$

where *N* is the batch size, *C* is the total number of classes, $y_{ni}$ is the actual probability of the *n*-th sample belonging to the *i*-th class (usually in one-hot encoded form), and ${\hat{y}}_{ni}$ is the predicted probability of the *n*-th sample belonging to the *i*-th class by the model.

In this experiment, Pixel Accuracy (PA), Mean Pixel Accuracy (MPA), Mean Intersection over Union (MIoU), and Frequency Weighted Intersection over Union (FWIoU) are selected as metrics. The specific formulas are as follows:

(9) $$PA=\frac{\sum_{i=1}^{C}TP_{i}+TN_{i}}{\sum_{i=1}^{C}TP_{i}+FP_{i}+FN_{i}}$$

(10) $$MPA=\frac{1}{C}\sum_{i=1}^{C}\frac{TP_{i}}{TP_{i}+FP_{i}+FN_{i}}$$

(11) $$MIoU=\frac{1}{C}\sum_{i=1}^{C}\frac{TP_{i}}{TP_{i}+FP_{i}+FN_{i}}$$

(12) $$FWIoU=\frac{\sum_{i=1}^{C}w_{i}\cdot \frac{TP_{i}}{TP_{i}+FP_{i}+FN_{i}}}{\sum_{i=1}^{C}w_{i}}$$

where $TP_{i}$ is the true positives, $TN_{i}$ is the true negatives, $FP_{i}$ is the false positives, and $FN_{i}$ is the false negatives for class *i*. *C* represents the total number of classes. $w_{i}$ represents the frequency of class *i* in the ground truth labels, calculated as $w_{i}=\frac{\sum_{j=1}^{N}1\left(y_{j}=i\right)}{N}$, where *N* denotes the total number of pixels, and $1(y_{j}=i)$ indicates 1 when the true label of pixel *j* is category *i*, and 0 otherwise.

### 4.3. Ablation Experiment

Since we use the U-shaped network to design MDU-Net, the ablation experiments allow us to replace the backbone network of the encoder and compare it with the original U-shaped structure. We use four metrics, frequency Weighted Intersection over Union (FWIoU), pixel accuracy (PA), mean Intersection over Union (MIOU), and mean new pixel accuracy (MPA) as the evaluation criteria for the experiments. The model depends on the design of the convolutional module to extract features, so we divide a segmentation model into an encoder–decoder structure and take the best metrics from these 100 training cycles.

As shown in Table 1, we divide the encoder into ordinary convolution module, residual module, multi-scale residual module, and its variants, while the encoder in addition to ordinary up-sampling also has our double up-sampling module. MultiResUNet is not very effective in the cloud shadow detection task, and the accuracy of MIOU is only 76.06%. DC-UNet [33] even directly performs two MultiResBlock operations on the input data separately and then sums them up, and the model effect is seriously degraded. In addition, MultiResBlock focuses too much on the reuse of densely connected features, and also has to control the number of channels of the output feature maps, resulting in the fact that the number of convolution kernels is only one-sixth to half of that of the ordinary convolution and the extracted feature maps contain seriously insufficient cloud layer texture information.

Compared to common convolution, the skip connection added by the residual module not only solves the model degradation problem of too deep network layers, but also better complements the low-level features such as color texture rich in clouds and shadows. Meanwhile, the residual block can better deepen these networks. UNet essentially only performs eight convolutional operations, and compared to ResNet18’s 18 convolutional down-sampling, the network is not deep enough, and the extracted high-level semantic information is also insufficient, so it is nearly 1 percentage point worse than ResNet34 in the MIOU metric. The total number of datasets for the cloud detection task is only 2557, which belongs to the small-sample task, and the experimental accuracy of using ResNet50 with too deep a network shows a decreasing trend, and the model is not able to extract a better representation of advanced features such as cloud contours. A suitable network depth can only help in cloud and shadow detection, so our MDU-Net chooses ResNet34 as its backbone network.

### 4.4. Comparison of Results in Complex Scenes

Since the main task of this paper is detecting cloud and cloud shadows in complex scenes, in this subsection, we use classical semantic segmentation models to predict these results and compare the strengths and weaknesses of the respective models by visualization images.

**Figure 5:**
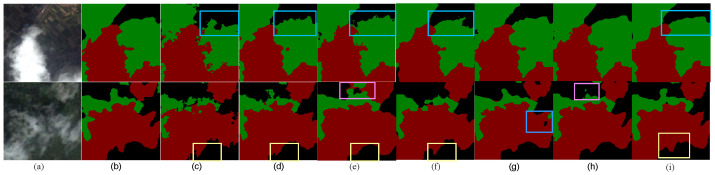
Visualization results of different models in rural and vegetated regions. (**a**) Original image, (**b**) Label image, (**c**) FCN, (**d**) UNet, (**e**) MultiResUNet, (**f**) PSPNet, (**g**) RSAGUNet, (**h**) AFMUNet, (**i**) MDU-Net.

Due to the dark colors of the black soil and vegetation, the background has a high probability of being misinterpreted as cloud shadows. At the same time, the cloud thickness of thin clouds is not thick enough to be easily confused with the background, which will often lead to missed detection. As shown in Figure 5, the blue boxes in the first row of the image are marked as the regions where obvious misclassification is detected. Our MDU-Net adopts the original features of the residual structure to retain the location and category information of the cloud shadows well, so as to locate the cloud shadows better and reduce the interference of dark backgrounds such as black land and vegetation.

Similarly, the yellow box in the second row of images circles the thin and broken clouds and vegetation (background) detection results. Due to the insufficient thickness of the thin clouds, confused with the vegetation in the background, the boundary range is very fuzzy, so the accurate positioning of small targets puts forward requirements on the model feature extraction ability. And the purple box in the second row of images highlights the problem of mixed messages also occurring at the junction of clouds, shadows, and background. As shown in the blue box in Figure 5g, RSAGUNet still fails to resolve the issue of confusion and misjudgment between thin clouds and dark backgrounds. In the purple box in Figure 5h, although AFMUNet has modified the feature fusion method, it still struggles to accurately extract features at the junctions where they mix.

MDU-Net extracts the cloud edge information, which not only eliminates the noise interference of the background in the image, but also accurately locates the position of clouds, shadows, and vegetation, which further reduces the possibility of omission and classification error.

The confusion of background with clouds and shadows occurs more often in darker scenes. When we process the cloud shadow dataset, we find that, under the cloud shadow, many bright features, such as saline, snow-covered, and thin broken clouds, cannot be detected accurately or ignored.

**Figure 6:**
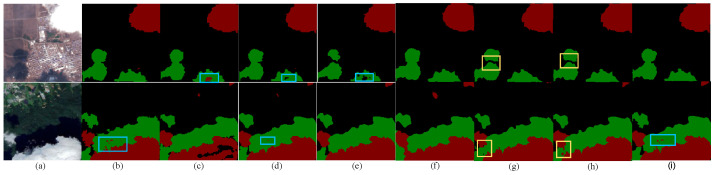
Prediction pictures of different algorithms in saline and snow-covered areas. (**a**) Original image, (**b**) Label image, (**c**) FCN, (**d**) UNet, (**e**) MultiResUNet, (**f**) PSPNet, (**g**) RSAGUNet, (**h**) AFMUNet, (**i**) MDU-Net.

As shown in Figure 6, the background of the first row of images is a combination of urban and rural areas with the presence of large areas of farmland. When the time of shooting is winter with a significant amount of snow cover, the background should be directly ignored even if it is under the shadow. However, from the labeled area in the blue box, FCN defaults them to clouds directly, while UNet and MultiResUNet recognize them as background. From the yellow boxes in Figure 6g,h, it can be seen that the feature fusion and residual modules of RSAGUNet and AFMUNet are insufficient for recognizing similar pixels, leading to mutual confusion between cloud shadows and the background.

In contrast, the full flow of contextual information module in MDU-Net achieves the fusion of high-level features rich in semantic information with low-level features rich in spatial information from a full-scale perspective, which allows the model to avoid the misinformation of the bright features under the shadows.

Covered by shadows are not only the bright-colored overlays as the background, but also the small targets such as thin and broken clouds that need to be extracted in this paper. As shown in the second row of images in Figure 6, scattered thin and broken clouds are covered by large cloud shadows, which we identify with blue boxes. MDU-Net directly extracts the feature maps of different stages for fusion in the second up-sampling module, which preserves the deep global information and the relationship between contextual positions, and achieves the detection of some thin and broken clouds on large cloud shadows.

### 4.5. Comparison of Results in General Scenes

In this subsection, MDU-Net is comprehensively quantitatively compared with other models by mixing complex and simple scene images. We present the mean accuracy results for four metrics. As shown in Table 2, MDU-Net outperforms classical semantic segmentation models on all four metrics.

Based on the above experimental results, we think that FCN ignores the spatial regularization step required by the semantic segmentation task based on pixel classification, which leads to the lack of spatial consistency of the results. The convolution module and network depth of UNet cannot meet the requirements of edge refinement of cloud features. The four characteristic images of PSPNet are all from the output of the same encoder, and they are only up-sampled once, which leads to incomplete fusion of context information.

In the encoder and decoder, ResUNet [34] replaces ordinary modules with residual modules and uses 3×3 convolutions in the branches instead of 1×1 convolutions. This not only increases the model’s parameters and computational load but also fails to retain the initial information of the input image, thereby reducing the refinement effect of the cloud shadow contour edges.

Compared to ResUNet, RSAGUNet [35] introduced a new attention fusion module during the skip connection phase. This module actively highlights key features while suppressing less important ones in cloud images. Therefore, RSAGUNet achieves scores of 94.77% in PA and 82.57% in MIOU, showing a slight superiority over ResUNet in all four metrics.

Recently, AFMUNet [36] designed a U-shaped structure that uses only conventional down-sampling modules in the encoder stage and introduces a channel-spatial attention module in the decoder stage. This module combines channel attention with spatial attention, selecting different channel feature maps to integrate richer contextual information and enhance the extraction of edge details in cloud shadows. Compared to RSAGUNet, AFMUNet’s newly designed feature fusion module replaces addition with concatenation before processing shallow and deep features, thus better preserving original information and improving detection accuracy. Therefore, AFMUNet’s performance is very close to that of our MDU-Net in all four metrics.

In comparion, our MDU-Net not only makes use of the appropriate residual module and network depth, but also dual up-sampling module to achieve the full flow of contextual information. For the task of detecting cloud masks in general scenes, MDU-Net achieves a MIOU result of 84.97% and already outperforms classical semantic segmentation models in other metrics as well.

### 4.6. Comparison on Other Dataset

In Section 4.6, we add robustness experiments on the landcover.ai dataset [37]. This dataset applies semantic segmentation to land cover classification and includes four types of surface features, where buildings are red, vegetation is green, water bodies are gray, and the background is black.

**Figure 7:**
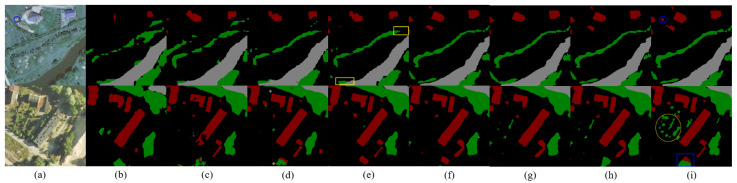
Visualization results of different models on landcover.ai dataset: (**a**) Original image, (**b**) FCN, (**c**) MultiResUNet, (**d**) ResUNet, (**e**) UNet, (**f**) PSPNet, (**g**) RSAGUNet, (**h**) AFMUNet, (**i**) MDU-Net.

As shown in Figure 7, in the first row of the visualization results, FCN, MultiResUNet, and ResUNet cannot accurately identify continuous vegetation areas. In the yellow box, water bodies are very close to vegetation but are not connected; however, UNet defaults to classifying this area as vegetation. Our model not only clearly distinguishes vegetation and water bodies but also detects buildings with minimal area, as shown by the blue boxes.

In the second row of the visual effects, within the blue box, MDU-Net continues to perform precise detection of connected features and confused categories. Moreover, due to lighting conditions, other models generally fail to segment out scattered trees in the area marked by the yellow box, whereas MDU-Net successfully does so. These accurate visualization results stem from the dual up-sampling module, which enables full circulation of contextual information, ensuring accurate contour positioning of surface features.

## 5. Conclusions

This paper propose an effective multiscale dual up-sampling network MDU-Net for cloud shadow detection in remote sensing images, which has an encoding–decoding–decoding structure. The encoder adopts ResNet34 as the backbone network. The decoder achieves full flow of contextual information between each pair of neighboring layers, ensuring that the feature maps extracted by the model focus on both local regions of the same layer and global information across layers. This not only strengthens the model’s advantage of accurate spatial localization for small targets such as thin and broken clouds, but also fulfills the task of detecting special scenes such as saline and snow in shadows.

However, our model still has some shortcomings. To recognize targets in complex scenes, ResNet34 and the dual up-sample module in fact increase parameters of the model, which also leads to an increase in inference speed as well as a decrease in FPS. In the future, under the premise of ensuring the accuracy of the model, we will adopt lightweight methods to solve the efficiency problem.

## Figures and Tables

**Table 1:** Ablation experiments of MDU-Net.

Model	PA (%)	MPA (%)	FWIoU (%)	MIoU (%)
UNet	95.41	93.42	89.79	83.86
MultiResBlock + DUM	91.70	88.94	84.88	76.06
DC Block + DUM	82.22	64.13	67.02	56.17
ResNet18 + DUM	95.36	94.85	91.20	84.27
ResNet34 + DUM	**95.61**	**94.81**	**91.52**	**84.97**
ResNet50 + DUM	95.07	93.56	89.65	83.62

Note: Bold indicates the maximum value of this metrics.

**Table 2:** Comparison of metrics.

Model	PA (%)	MPA (%)	FWIoU (%)	MIoU (%)
FCN [1]	93.92	93.36	88.51	81.09
UNet [2]	95.41	93.42	89.79	83.86
PSPNet [3]	95.48	94.53	91.16	84.08
MultiResUNet [30]	94.39	91.65	88.48	79.52
ResUNet [34]	94.31	91.69	87.43	81.44
RSAGUNet [35]	94.77	92.15	88.96	82.57
AFMUNet [36]	95.59	94.63	91.35	84.49
MDU-Net	**95.61**	**94.81**	**91.52**	**84.97**

Note: Bold indicates the maximum value of this metric in the original table.

## Data Availability

The data presented in this study are available on request from the corresponding author.

## References

[1] Long J., Shelhamer E., Darrell T. Fully convolutional networks for semantic segmentation. Proceedings of the IEEE Conference on Computer Vision and Pattern Recognition.

[2] Ronneberger O., Fischer P., Brox T. (2015). U-net: Convolutional networks for biomedical image segmentation. Medical Image Computing and Computer Assisted Intervention MICCAI 2015, Proceedings of the 18th International Conference, Munich, Germany, 5–9 October 2015.

[3] Zhao H., Shi J., Qi X., Wang X., Jia J. Pyramid scene parsing network. Proceedings of the IEEE Conference on Computer Vision and Pattern Recognition.

[4] Lin F., Liang Z., Wu S., He J., Chen K., Tian S. (2023). Structtoken: Rethinking semantic segmentation with structural prior. IEEE Trans. Circuits Syst. Video Technol..

[5] Cong W., Cong Y., Dong J., Sun G., Ding H. (2023). Gradient-semantic compensation for incremental semantic segmentation. IEEE Trans. Multimed..

[6] Rafi T.H., Mahjabin R., Ghosh E., Ko Y.W., Lee J.G. (2024). Domain generalization for semantic segmentation: A survey. Artif. Intell. Rev..

[7] Liu Y., Bai X., Wang J., Li G., Li J., Lv Z. (2024). Image semantic segmentation approach based on DeepLabV3 plus network with an attention mechanism. Eng. Appl. Artif. Intell..

[8] Sung C., Kim W., An J., Lee W., Lim H., Myung H. Contextrast: Contextual Contrastive Learning for Semantic Segmentation. Proceedings of the IEEE/CVF Conference on Computer Vision and Pattern Recognition.

[9] Thisanke H., Deshan C., Chamith K., Seneviratne S., Vidanaarachchi R., Herath D. (2023). Semantic segmentation using Vision Transformers: A survey. Eng. Appl. Artif. Intell..

[10] Lu J., Wang Y., Zhu Y., Ji X., Xing T., Li W., Zomaya A.Y. (2019). P_SegNet and NP_SegNet: New neural network architectures for cloud recognition of remote sensing images. IEEE Access.

[11] He K., Zhang X., Ren S., Sun J. Deep residual learning for image recognition. Proceedings of the IEEE Conference on Computer Vision and Pattern Recognition.

[12] Chen G., E D. (2007). Support vector machines for cloud detection over ice-snow areas. Geo-Spat. Inf. Sci..

[13] Fu H., Shen Y., Liu J., He G., Chen J., Liu P., Qian J., Li J. (2018). Cloud detection for FY meteorology satellite based on ensemble thresholds and random forests approach. Remote Sens..

[14] Liu C., Yang S., Di D., Yang Y., Zhou C., Hu X., Sohn B.J. (2022). A machine learning-based cloud detection algorithm for the Himawari-8 spectral image. Adv. Atmos. Sci..

[15] Ibrahim E., Jiang J., Lema L., Barnabé P., Giuliani G., Lacroix P., Pirard E. (2021). Cloud and cloud-shadow detection for applications in mapping small-scale mining in Colombia using sentinel-2 imagery. Remote Sens..

[16] Miroszewski A., Mielczarek J., Czelusta G., Szczepanek F., Grabowski B., Le Saux B., Nalepa J. (2023). Detecting clouds in multispectral satellite images using quantum-kernel support vector machines. IEEE J. Sel. Top. Appl. Earth Obs. Remote Sens..

[17] Gao X., Chi H., Huang J., Han Y., Li Y., Ling F. (2024). Comparison of Cloud-Mask Algorithms and Machine-Learning Methods Using Sentinel-2 Imagery for Mapping Paddy Rice in Jianghan Plain. Remote Sens..

[18] Yu X., Lary D.J. (2021). Cloud Detection Using an Ensemble of Pixel-Based Machine Learning Models Incorporating Unsupervised Classification. Remote Sens..

[19] Krizhevsky A., Sutskever I., Hinton G.E. Imagenet classification with deep convolutional neural networks. Proceedings of the Advances in Neural Information Processing Systems 25: 26th Annual Conference on Neural Information Processing Systems 2012.

[20] Simonyan K., Zisserman A. (2014). Very deep convolutional networks for large-scale image recognition. arXiv.

[21] Liu S., Chen P., Zhang Y. (2023). A multi-scale feature pyramid SAR ship detection network with robust background interference. IEEE J. Sel. Top. Appl. Earth Obs. Remote Sens..

[22] Li J., Wang Q. (2024). CSDFormer: A cloud and shadow detection method for landsat images based on transformer. Int. J. Appl. Earth Obs. Geoinf..

[23] Dong J., Wang Y., Yang Y., Yang M., Chen J. (2024). MCDNet: Multilevel cloud detection network for remote sensing images based on dual-perspective change-guided and multi-scale feature fusion. Int. J. Appl. Earth Obs. Geoinf..

[24] Zhang G., Gao X., Yang J., Yang Y., Tan M., Xu J., Wang Y. (2022). A multi-task driven and reconfigurable network for cloud detection in cloud-snow coexistence regions from very-high-resolution remote sensing images. Int. J. Appl. Earth Obs. Geoinf..

[25] Zhang C., Weng L., Ding L., Xia M., Lin H. (2023). CRSNet: Cloud and cloud shadow refinement segmentation networks for remote sensing imagery. Remote Sens..

[26] Chen K., Dai X., Xia M., Weng L., Hu K., Lin H. (2023). MSFANet: Multi-scale strip feature attention network for cloud and cloud shadow segmentation. Remote Sens..

[27] Gu G., Weng L., Xia M., Hu K., Lin H. (2024). Muti-path Muti-scale Attention Network for Cloud and Cloud shadow segmentation. IEEE Trans. Geosci. Remote Sens..

[28] Jeppesen J.H., Jacobsen R.H., Inceoglu F., Toftegaard T.S. (2019). A cloud detection algorithm for satellite imagery based on deep learning. Remote Sens. Environ..

[29] Jiang B., Li X., Chong H., Wu Y., Li Y., Jia J., Wang S., Wang J., Chen X. (2022). A deep-learning reconstruction method for remote sensing images with large thick cloud cover. Int. J. Appl. Earth Obs. Geoinf..

[30] Ibtehaz N., Rahman M.S. (2020). MultiResUNet: Rethinking the U-Net architecture for multimodal biomedical image segmentation. Neural Netw..

[31] Du X., Wu H. (2024). Gated aggregation network for cloud detection in remote sensing image. Vis. Comput..

[32] Wang Q., Blackburn G.A., Onojeghuo A.O., Dash J., Zhou L., Zhang Y., Atkinson P.M. (2017). Fusion of Landsat 8 OLI and Sentinel-2 MSI data. IEEE Trans. Geosci. Remote Sens..

[33] Lou A., Guan S., Loew M. (2021). DC-UNet: Rethinking the U-Net architecture with dual channel efficient CNN for medical image segmentation. Proceedings of the Medical Imaging 2021: Image Processing.

[34] Diakogiannis F.I., Waldner F., Caccetta P., Wu C. (2020). ResUNet-a: A deep learning framework for semantic segmentation of remotely sensed data. Isprs J. Photogramm. Remote Sens..

[35] Kumar A., Kashyap Y., Divakar P. (2024). ResAG-UNet: A Novel Residual Attention Gated UNet for Cloud Segmentation in Sky Image. IEEE J. Photovoltaics.

[36] Du W., Fan Z., Yan Y., Yu R., Liu J. (2024). AFMUNet: Attention Feature Fusion Network Based on a U-Shaped Structure for Cloud and Cloud Shadow Detection. Remote Sens..

[37] Boguszewski A., Batorski D., Ziemba-Jankowska N., Zambrzycka A., Dziedzic T. (2020). Landcover. ai: Dataset for automatic mapping of buildings, woodlands and water from aerial imagery. arXiv.

